# Persistent SARS-CoV-2 infection in patients with secondary antibody deficiency: successful clearance following combination casirivimab and imdevimab (REGN-COV2) monoclonal antibody therapy

**DOI:** 10.1186/s12941-021-00491-2

**Published:** 2021-12-30

**Authors:** Yusri Taha, Hayley Wardle, Adam B. Evans, Ewan R. Hunter, Helen Marr, Wendy Osborne, Matthew Bashton, Darren Smith, Shirelle Burton-Fanning, Matthias L. Schmid, Christopher J. A. Duncan

**Affiliations:** 1 Department of Infectious Diseases and Tropical Medicine, The Newcastle Upon Tyne Hospitals NHS Foundation Trust, Newcastle upon Tyne, UK; 2 Microbiology and Virology Department, Laboratory Medicine Directorate, The Newcastle Upon Tyne Hospitals NHS Foundation Trust, Newcastle upon Tyne, UK; 3 Department of Haematology, The Newcastle Upon Tyne Hospitals NHS Foundation Trust, Newcastle upon Tyne, UK; 4 The Hub for Biotechnology in the Built Environment, Department of Applied Sciences, Faculty of Health and Life Sciences, Northumbria University, Newcastle upon Tyne, UK; 5 Translational and Clinical Research Institute, Immunity and Inflammation Theme, Newcastle University, Newcastle upon Tyne, UK; 6 Faculty of Medical Sciences, Leech Building, Newcastle University Medical School, Room M3. 119, 3rd Floor, Newcastle upon Tyne, NE2 4HH UK

**Keywords:** Antibody deficiency, Primary and secondary immunodeficiency, Chronic COVID-19, Passive immunisation, Ronapreve (REGN-COV2), B cell depleting therapy, Omicron

## Abstract

**Background:**

There is growing evidence that antibody responses play a role in the resolution of SARS-CoV-2 infection. Patients with primary or secondary antibody deficiency are at increased risk of persistent infection. This challenging clinical scenario is associated with adverse patient outcome and potentially creates an ecological niche for the evolution of novel SARS-CoV-2 variants with immune evasion capacity. Case reports and/or series have implied a therapeutic role for convalescent plasma (CP) to secure virological clearance, although concerns have been raised about the effectiveness of CP and its potential to drive viral evolution, and it has largely been withdrawn from clinical use in the UK.

**Case presentation:**

We report two cases in which persistent SARS-CoV-2 infection was cleared following administration of the monoclonal antibody combination casirivimab and imdevimab (REGN-COV2, Ronapreve). A 55-year-old male with follicular lymphoma, treated with B cell depleting therapy, developed SARS-CoV-2 infection in September 2020 which then persisted for over 200 days. He was hospitalised on four occasions with COVID-19 and suffered debilitating fatigue and malaise throughout. There was no clinical response to antiviral therapy with remdesivir or CP, and SARS-CoV-2 was consistently detected in nasopharyngeal swabs. Intrahost evolution of several spike variants of uncertain significance was identified by viral sequence analysis. Delivery of REGN-COV2, in combination with remdesivir, was associated with clinical improvement and viral clearance within 6 days, which was sustained for over 150 days despite immunotherapy for relapsed follicular lymphoma. The second case, a 68-year-old female with chronic lymphocytic leukaemia on ibrutinib, also developed persistent SARS-CoV-2 infection. Despite a lack of response to remdesivir, infection promptly cleared following REGN-COV2 in combination with remdesivir, accompanied by resolution of inflammation and full clinical recovery that has been maintained for over 290 days.

**Conclusions:**

These cases highlight the potential benefit of REGN-COV2 as therapy for persistent SARS-CoV-2 infection in antibody deficient individuals, including after failure of CP treatment. Formal clinical studies are warranted to assess the effectiveness of REGN-COV2 in antibody-deficient patients, especially in light of the emergence of variants of concern, such as Omicron, that appear to evade REGN-COV2 neutralisation.

## Background

The recognition that SARS-CoV-2 infection leads to a degree of natural immunity to reinfection [1–3], allied to the development of several highly effective vaccines [4–7], is cause for optimism that the impact of the COVID-19 pandemic might ultimately lessen. Whilst the specific immunological mechanisms underpinning immunity remain to be defined [8], SARS-CoV-2 binding and/or neutralising antibodies appear to correlate with protection against infection or reinfection [1–3, 8–11]. It is also becoming apparent that patients with primary or secondary defects of humoral immunity exhibit suboptimal responses to natural infection and/or vaccination and are susceptible to persistent or chronic SARS-CoV-2 infection [12–28], implicating antibodies in the resolution of COVID-19.

Beyond the clinical importance of chronic SARS-CoV-2 infection and its associated morbidity for the individual concerned, persistently infected hosts may provide a habitat for the emergence of viral variants of concern with the capacity to transmit more efficiently and/or evade immunity, representing a potential risk to public health and infection control [29]. Treatment strategies for persistent infection are therefore needed [29] and one potential solution is immunotherapy, via the transfer of functional antibody to a seronegative recipient [30]. Options to deliver this include either convalescent plasma (CP) harvested from immunocompetent individuals following recovery from COVID-19, or specifically engineered recombinant neutralising monoclonal antibody (mAb) preparations [30]. Whilst clinical trials of CP as a treatment for patients hospitalised with COVID-19 failed to show benefit in a predominantly immunocompetent patient cohort [31], leading to its withdrawal from clinical use, favourable responses to CP have been reported in case reports and case series of patients with antibody deficiency (reviewed in [29]). Nevertheless, there are also reports of a lack of response to CP [15, 19], with the unintended consequence of driving evolution of novel variants. Randomised trials of mAb preparations have reported efficacy in ambulatory patients [32] and unpublished data from the RECOVERY trial indicate efficacy in seronegative hospitalised patients. However to date, relatively few published reports describe the response to mAb therapy in antibody-deficient patients, and none to our knowledge have reported its performance as salvage therapy following failure of CP.

## Case report

### Case 1

This 55-year-old male with an 8-year history of stage IV follicular lymphoma, with extranodal disease involving pleura and peritoneal effusions, first tested positive for SARS-CoV-2 on a nasopharyngeal swab taken for asymptomatic screening on 14 September 2020. The patient had previously received multiple courses of prior chemotherapy including rituximab, cyclophosphamide, doxorubicin, vincristine and prednisolone (R-CHOP) with rituximab maintenance. He was subsequently switched to glofitamab (anti-CD20/anti-CD3 bispecific antibody) and atezolizumab (anti-PD-L1) via a clinical trial and achieved remission with an excellent performance status. Other significant past medical history included non-insulin dependent diabetes mellitus (NIDDM) treated with metformin and insulin, and previous pulmonary embolism.

**Fig. 1:**
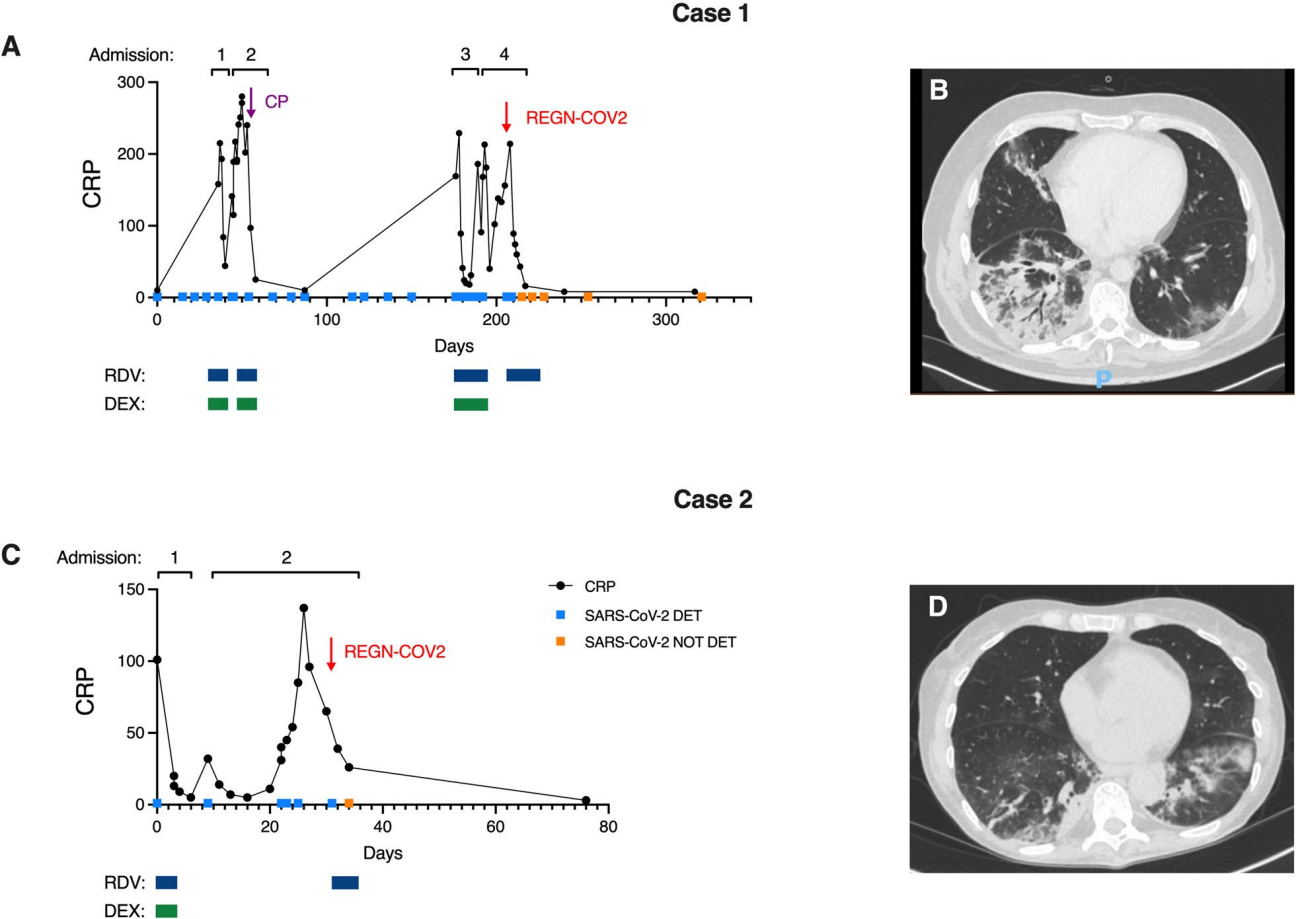
CRP, SARS-CoV-2 PCR testing results and timeline of therapies in Cases 1 (**A**) and 2 (**C**). Light blue squares represent positive and orange squares negative swab PCR results. Dark blue and green boxes demonstrate treatment episodes of remdesivir (RDV) and dexamethasone (DEX) respectively. Purple arrow represents convalescent plasma (CP) administration (given over two days) and red arrows casirivimab and imdevimab (REGN-COV2) administration. CT thorax appearances prior to RGN therapy in Cases 1 (**B**) and 2 (**D**) demonstrating pneumonitis

Despite prompt discontinuation of glofitamab and atezolizumab he remained PCR positive on three occasions over the following month and was admitted to hospital on 20 October 2020 after developing symptoms of COVID-19 (fever, rigors and breathing difficulties). On admission he was febrile (39.1 °C) and hypoxic (SpO2 91% on room air) and he received the local standard treatment for severe COVID-19 (oxygen, dexamethasone 6 mg od PO, remdesivir 100 mg od IV). Investigations revealed elevated CRP (158 mg/L, Fig. 1A), persistent detection of SARS-CoV-2 (Table 1) and radiographic changes consistent with COVID-19 pneumonitis. He was discharged four days later after an initially favourable clinical response, but was readmitted after another four days with ongoing fever. CT thorax identified changes consistent with COVID-19 pneumonitis (Fig. 1B) and serological testing for SARS-CoV-2 nucleocapsid antibody was negative, suggesting a failure of seroconversion at this stage. Management after readmission included broad-spectrum antibiotic and antifungal therapy (initially piperacillin-tazobactam, subsequently meropenem and caspofungin). Due to a lack of response to antibiotic therapy, he was re-treated with dexamethasone 6 mg od PO and remdesivir 100 mg od IV (both for 5 days). In addition, CP—obtained from NHS Blood and Transplant—was administered in two infusions (at 54 and 55 days after SARS-CoV-2 infection was initially identified), with an immediate apparent clinical benefit (subjective improvement in symptoms, resolution of fever, and reduction in CRP, Fig. 1A). Admission on this occasion was for 6 days.

**Table 1:** Virological analyses, case 1

Days post infection	SARS-CoV-2 PCR	Assay used	Mean C_T_ value	SARS-CoV-2 N Ab (units)
0	Detected	1	23.1	–
15	Detected	1	22.2	–
22	Detected	1	28.4	–
29	Detected	2	26.3	–
36	Detected	2	29.3	–
44	Detected	2	24.1	–
52	–		–	Not detected (0.09)
54	Detected	1	28.2	–
55	–		–	Detected (2.07)
63	–		–	Detected (13.19)
68	Detected	1	27.6	-
79	Detected	1	29.3	Detected (5.74)
87	Detected	1	27.9	–
115	Indeterminate	3	28.5	–
122	Detected	3	26.9	–
136	Detected	2	32.8	Detected (1.17)
150	Detected	2	23.9	Not detected (0.78)
176	Detected	4	–	–
182	Detected	2	32.8	–
185	Detected	2	31.9	–
189	Detected	4	–	–
190	Detected	1	23.5	–
206	Detected	2	31.7	–
208	Detected	1	28.7	–
213	Not detected	1	–	–
215	Not detected	1	–	–
217	-		–	Not detected
221	Not detected	2	–	–
228	Not detected	1	–	–
254	Not detected	1	–	–
321	Not detected	2	–	–

PCR assay and gene targets: [Altona RealStar® SARS-CoV-2 RT-PCR Kit 1.0 (S & E); 2. Roche: Cobas® SARS-CoV-2 Test (Orf1a/b & E); 3. Mobiag: Amplidiag® COVID-19 (Orf1a/b & N); 4. DRW: SAMBA II SARS-CoV-2 Test (Orf1a/b & N)]^.^ Antibody assay: [Roche Elecsys Anti-SARS-CoV-2 assay (nucleocapsid). CP was administered on day 54 and REGN-CoV2 at day 207

Upon review in outpatient clinic approximately four weeks later the patient continued to experience ongoing symptoms, including debilitating malaise, fatigue and a substantial reduction in exercise tolerance. Although CRP had fallen to 10, SARS-CoV-2 continued to be detectable in upper respiratory tract samples, and interval CT scanning revealed persistent lung changes consistent with COVID-19, along with a significant increase in lymphadenopathy, a right-sided pleural effusion and splenomegaly, suggestive of relapse of FL. Owing to the apparent progression of lymphoma and persistent SARS-CoV-2 which precluded further immunotherapy, he was hospitalised again on 9 March 2021 for further management, 176 days from the first isolation of SARS-CoV-2, at which point CRP was elevated again (163). He received further treatment with dexamethasone 6 mg od PO and remdesivir 100 mg od IV, this time for 10 days. In addition, off-label treatment with nitazoxanide 500 mg tds PO for 7 days [33] was started and he was discharged to complete this as an outpatient. He was then admitted for a fourth time three days later feeling generally unwell. Nitazoxanide was discontinued after a total of seven days’ treatment and attempts were made to source monoclonal antibody therapies for compassionate use with the aim of achieving viral clearance. When this proved not to be possible, the patient was recruited into the RECOVERY trial and randomised to receive REGN-COV2 (4 g casirivimab and 4 g imdevimab), which was administered at day 207 after initial detection of the virus, along with a further concomitant course of remdesivir (100 mg od IV for 10 days). CRP settled rapidly, in this case without administration of dexamethasone, and the patient was discharged to complete parenteral remdesivir on an outpatient basis. During this period the patient also received two doses of AZD1222 COVID-19 vaccine, on day 193 (prior to REGN-COV2) and 286.

A notable feature of the case was the continued detection of SARS-CoV-2 RNA by clinical RT-PCR testing (for assays see Table 1). The cycle threshold (C_T_) value fluctuated between ~ 22–32 for 6 months, with no apparent evidence of a reduction in viral titre (which would be reflected in a rise in C_T_) following the initial course of remdesivir treatment on admission 1 or following remdesivir and CP on admission 2 (Table 1). However, owing to different assay platforms used for PCR analysis, direct comparison of C_T_ values may not be reliable. SARS-CoV-2 was not detected in blood on two occasions day 185 and 208. Interestingly, SARS-CoV-2 became undetectable in nasopharyngeal swabs when first tested 6 days after administration of REGN-COV2. Ongoing screening of nasopharyngeal swabs has not identified SARS-CoV-2 up to 150 days post-REGN-COV2 despite further bispecific antibody therapy, glofitamab, for relapsed follicular lymphoma (Fig. 1A, Table 1).

Immunological investigations demonstrated hypogammaglobulinaemia associated with initial B cell lymphopenia, the latter recovering during the period of SARS-CoV-2 infection following withdrawl of glofitamab (Table 2). Analysis of nucleocapsid (N) antibody titre in serum was also undertaken to assess specific antibody production (Table 1). This demonstrated the absence of N antibodies 52 days after infection. Following administration of CP there was a modest rise in N binding IgG in serum, which waned after approximately 100 days, in keeping with passive immunisation. The patient remained N seronegative at day 150, and again at day 217. Collectively these data indicated a defective antibody response to SARS-CoV-2 infection.

**Table 2:** Immunophenotyping in case 1

Parameter	Pre-COVID-19	Pre-RGN	Post-RGN	Normal
IgA (g/L)	0.69	0.74	**0.44**	0.64–2.97
IgG (g/L)	**4.6**	**3.7**	**3.1**	5.8–15.4
IgM (g/L)	**0.05**	**0.06**	**0.07**	0.24–1.90
CD3 + (cells/µL)	1030	1017	N/D	690–2540
CD19 + (cells/µL)	0	360	N/D	90–660
CD16 + /CD56 + (cells/µL)	163	168	N/D	90–590
CD3 + /CD4 + (cells/µL)	N/D	345	N/D	410–1590
CD3 + /CD8 + (cells/µL)	N/D	677	N/D	190–1140
Lymphocytes (cells/µL)	1204	1552	N/D	N/A

The absence of B cells (CD19 +) prior to COVID-19 reflects treatment with glofitamab and prior therapy with rituximab. Although B cell numbers recovered following discontinuation of glofitamab, hypogammaglobulinaemia persisted

*N/D* not done, *N/A* not applicable

Viral sequence analysis was undertaken separately, as part of the COVID-19 Genomics UK Consortium (COG-UK) study, on nasopharyngeal samples obtained at days 29, 79 and 150 post infection. The results are summarised in Table 3. All sequences were identified as European B.1.389 lineage, with no suggestion of superinfection. Analysis of the initial sample (day 29) revealed two missense variants in spike gene – D614G and T723I – characteristic of the B.1.389 lineage with no major changes in the sample from day 79. However the sample from day 150 demonstrated two additional spike protein changes—a missense G769A change in the downward helix and a deletion of 141–143 in the N-terminal domain (NTD). Functional testing of the significance of these variants has not yet been undertaken.

**Table 3:** Acquisition SARS-CoV-2 *spike* variants over time in case 1, as detected by whole genome sequencing

Day	Variant(s)			
29	A1841G; p.D614G	C2168T;p.T723I		
79	A1841G; p.D614G	C2168T;p.T723I		
150	A1841G; p.D614G	C2168T;p.T723I	419_427del; p.L141_V143del	G2306C; p.G769A

CP was administered at day 54 and REG-COV2 at day 207

### Case 2

A 68-year-old female with an 11-year history of chronic lymphocytic leukaemia (CLL) and secondary antibody deficiency, treated with the Bruton’s tyrosine kinase inhibitor ibrutinib, was hospitalised with hypoxaemia, fever and respiratory symptoms, having previously been identified as a contact of SARS-CoV-2. Previous complications of CLL included post-splenectomy pulmonary thromboembolism and recurrent autoimmune haemolytic anaemia. Other past medical history included traumatic retroperitoneal haemorrhage, depression and osteoporosis. On admission, SARS-CoV-2 was detected on a nasopharyngeal swab, CRP and procalcitonin were elevated (101 mg/L and 3.17 ng/mL respectively) (Fig. 1C) and chest radiograph revealed changes consistent with COVID-19. Treatment for severe COVID-19 was administered consisting of oxygen, dexamethasone 6 mg od PO and remdesivir 100 mg od IV. In addition, empirical antibiotics (piperacillin-tazobactam and clarithromycin) were prescribed to cover the possibility of community acquired pneumonia. The patient made an apparent response to treatment and was discharged on day 7. However she was promptly readmitted on day 9 with malaise, fever, raised inflammatory markers, and hypoxaemia. Empirical antibiotics were restarted, although multiple cultures of blood and urine were subsequently sterile. CT thorax demonstrated persistent changes suggestive of COVID-19 (Fig. 1D). Owing to ongoing fever despite broad-spectrum antibiotic therapy, caspofungin was added, but with no discernible clinical response. Virological investigations revealed persistent detection of SARS-CoV-2 in nasopharyngeal swabs (C_T_ ~ 22) and seronegativity for N antibody at 23 days post initial detection of virus, suggestive of a defect of specific antibody response. Consistent with this, immunoglobulin analysis three weeks prior to admission had demonstrated hypogammaglobulinaemia (IgA 0.25 g/L [0.64–2.97], IgG 2.3 g/L [5.8–15.4], IgM 0.40 [0.71–2.30]). Extended PCR analysis of respiratory secretions identified no evidence of viral or bacterial coinfection. On day 32 of admission, the patient was recruited to the RECOVERY trial and randomised to receive REGN-COV2, administered at day 32 (4 g casirivimab and 4 g imdevimab) alongside remdesivir 100 mg od IV for 5 days. CRP settled rapidly, without administration of dexamethasone, SARS-CoV-2 became undetectable on respiratory tract swabs on day 35, and the patient was discharged on day 37. Ibrutinib was restarted post discharge and there has been no recurrence of SARS-CoV-2 infection out to day 324.

## Discussion and conclusions

This report of prolonged SARS-CoV-2 infection with COVID-19 in two patients with a defective humoral response to SARS-CoV-2, evidenced by hypogammaglobulinaemia and the absence of detectable nucleocapsid binding IgG, reinforces the importance of antibodies in clearance of SARS-CoV-2 infection. Several reports are now available describing a prolonged course of COVID-19 in immunocompromised patients, largely in the context of primary or acquired antibody deficiency [12–28, 34, 35]. The clinical course in our patients was consistent with other reports, with episodes of hospitalisation with typical COVID-19 illness—featuring hypoxia, radiological evidence of pneumonitis and signs of inflammation—on a background of persistent debilitating symptoms of fever, fatigue, dyspnoea and malaise. Interestingly, these latter symptoms resolved promptly upon viral clearance, implying that they were predominantly driven by ongoing viral infection. This latter aspect highlights the clinical rationale for excluding ongoing viral infection in immunocompromised patients with prolonged ‘post-COVID-19’ symptoms.

We describe evidence of therapeutic efficacy of casirivimab and imdevimab (REGN-COV2) leading to clearance of persistent infection in these immunocompromised patients. Of particular note in Case 1, this was achieved despite a prior failure of CP therapy. The response to REGN-COV2 was evident in the rapid and sustained loss of viral RNA (vRNA) from the upper respiratory tract, accompanied by resolution of clinical symptoms and systemic inflammation, leading to full clinical recovery—all in close temporal association to the receipt of REGN-COV2. Given that unremitting clinical illness and detectable SARS-CoV-2 vRNA prior to exposure to REGN-COV2 had persisted for over 190 days in Case 1 and 32 days in Case 2, we postulate a causal association. The seminal randomised controlled trial of REGN-COV2 reported efficacy in the management of patients with COVID-19 in the outpatient setting, although did not report specifically on immuncompromised patient groups (patients on immunoglobulin supplementation were excluded) [32]. Other mAb therapies—e.g. bamlanivimab together with etesevimab, and sotrovimab—are also approved under emergency use authorisation in the US (reviewed in [30]). Preliminary unpublished results of the RECOVERY study demonstrate significant efficacy of REGN-COV2 in seronegative, but not seropositive hospitalised patients with COVID-19. In this study, seronegative patients had twice the baseline mortality rate of seropositive patients (30% vs 15%). A limited number of case reports have described therapeutic efficacy of mAb therapies in immunocompromised patient groups, however those that do are generally consistent with our observations. Luitel and colleagues described a patient with hypogammaglobulinaemia, treated with immunoglobulin replacement, who had a clinical response to REGN-COV2 monotherapy [27]. Interestingly, this patient had evidence of vRNA in bronchoalveolar lavage which was absent from the upper respiratory tract. Nguyen and colleagues reported effectiveness of REGN-COV2 in combination with remdesivir in a patient with X-linked agammaglobulinaemia (XLA) and persistent COVID-19 [23]. Conversely, Choi and colleagues reported use of REGN-COV2 in a rituximab-treated patient who was critically ill with COVID-19 and aspergillus coinfection and died 11 days after administration [36]. Finally, Kavanagh Williamson and colleagues reported a patient with hypogammaglobulinaemia secondary to chronic lymphocytic leukaemia in whom chronic infection for 290 days was successfully cleared by REGN-COV2 monotherapy [28]. Interestingly, the kinetic of resolution appeared relatively slow—taking 45 days, as opposed to 3 days in our case—although in the former the possibility of coinfection with B.1.1.7 lineage occurring after REGN-COV2 could not be excluded. While our report was under review, Brown and colleagues published a retrospective case series of patients with primary or secondary antibody deficiency, in which a 13/14 patients receiving either REGN-COV2 or CP with remdesivir (8/8 and 5/6 respectively) cleared the virus [34]. Collectively these findings are promising and support the argument for further studies of the clinical effectiveness of mAb therapy in antibody-deficient patient cohorts. It is also worth noting that doses used in the studies above were generally higher than the 2.4 g dose selected for clinical deployment in UK practice.

Responses to CP have been more widely reported in the literature. Generally these reports demonstrated effectiveness in immunocompromised patients [17, 18, 20–22, 34, 37]. Less frequently, CP failed to clear infection [15, 19], as in Case 1. In these reports, there were also temporal associations between administration of CP and the accumulation of viral mutations, suggesting that suboptimal antibody pressure may have driven viral evolution [15, 19]. This is clearly a cause for concern both from the perspective of the individual patient and for wider public health, arguing for caution in the use of CP in such patients. However in Case 1, viral sequencing provided inconclusive evidence of an association between CP administration and the acquisition of additional variants, since additional spike variants were only detected at three months but not at one month following CP receipt. The del141-143LGV and G769A variants map to the N-terminal domain (NTD) and downward helix of spike respectively. We note similarity of the former to the recurrent 141-144LGVY deletion, a ‘recurrent deletion’ region of NTD [38]. This is reported in immunocompromised patients [15] and is associated with a reduction in nAb efficacy [38]. The significance of G769A is not yet known. Although several factors may have accounted for the lack of efficacy of CP in our case and in other published cases, it is also consistent with the lack of efficacy of CP in hospitalised patients in large scale randomised clinical trials such as the RECOVERY study [31]. One factor that may influence the effectiveness of CP is the variable quantity of SARS-CoV-2 spike binding and/or neutralising antibody present within the plasma product. Information on the nAb content of the CP preparation used in patient 1 was not available. Arguably, this concern might also apply to a larger group of patients receiving regular antibody replacement therapy for primary or secondary antibody deficiency, in which variable nAb content of Ig products could theoretically exert selection pressure on SARS-CoV-2, leading to the evolution of variants of concern. An advantage of REGN-COV2, or other highly neutralising monoclonal antibody (mAb) products, is that the antibody content is consistent and well-defined, however the effective dose remains to be defined. Interestingly, in our cases there was limited evidence of a clinical or virological response to remdesivir in vivo. Similar findings have been observed elsewhere [23, 28], in situations where in vitro sensitivity of the SARS-CoV-2 isolate has been demonstrated [28], although in other reports a response to remdesivir was seen, albeit followed by viral recrudescence [14, 19, 36, 39, 40]. It remains an open question whether remdesivir is needed as an adjunct to REGN-COV2 or other mAb therapies in the management of chronic COVID-19 in immunocomromised patients. However, an important issue is the evolution of viral variants of concern that may undermine the therapeutic effectiveness of current mAb therapies. Recently a new variant of concern has emerged—B1.1.529 (Omicron)—which possesses a range of missense substitutions and indels in the spike receptor binding domain [41]. These mutations could significantly compromise efficacy of mAb therapies. Whilst data on in vivo effectiveness against Omicron are awaited, unpublished in vitro studies suggest that it completely evades casirivimab/imdevimab neutralisation. If this finding is reproducible and applicable to other mAbs, these will be rendered ineffective and would need to be redesigned if, as expected, Omicron comes to dominate worldwide.

In both cases reported here, resolution of inflammation occurred following viral clearance in the absence of steroid therapy. This suggests that sustained viral replication makes a relevant contribution to the inflammatory response underpinning COVID-19. Thus while immunomodulation with agents such as dexamethasone and/or tocilizumab is a logical strategy in immunocompetent patients, supported by clear evidence from randomised controlled trials [42, 43], there may also be a clinical need for antiviral therapies and/or interventions designed to enhance antiviral responses in immunocompromised patients. This is relevant since patients with pre-existing immunocompromise are at increased risk of infectious complications of agents such as corticosteroids or anticytokine therapies [23].

We acknowledge there are some important limitations to this report. In both cases, due to the co-administration of remdesivir, we were unable to definitively prove that resolution occurred solely as a consequence of REGN-COV2, although the absence of a virological response to previous courses of remdesivir along with the reported effectiveness of REGN-COV2 monotherapy [27, 28], suggests it is likely that REGN-COV2 made the dominant contribution. T-cell responses may provide a degree of protection against progression to severe disease in antibody-deficient patients [14]. We did not formally assess SARS-CoV-2-specific T-cell responses in these patients, although we documented normal numbers of T-cell subsets in Case 1 and the efficacy of CD20/CD3 bispecific therapy in lymphoma was evidence of a functional antitumour T cell response. Finally, whilst we did not undertake sub-genomic RNA PCR analysis or viral culture to prove definitively that persistent detection of viral RNA reflected ongoing replication, this is nevertheless a reasonable assumption, particularly given the rapid clearance following REGN-COV2.

In summary, we report two cases of persistent SARS-CoV-2 infection in antibody-deficient patients that were apparently cleared rapidly following administration of REGN-COV2. These cases highlight the potential benefit of REGN-COV2 in therapy for persistent SARS-CoV-2 infection in antibody-deficient individuals, including following failure of CP treatment, although with the caveat that clinical efficacy against Alpha or Delta variants does not imply efficacy against Omicron. In addition to urgently assessing therapeutic efficacy against Omicron, studies are warranted to assess the clinical effectiveness of mAb therapy in patients who are unable to generate functional antibodies, to clarify the dosage needed, to assess the value of antiviral coadministration, and to establish the potential risk of variant development if therapy is ineffective. Future studies should also continue to determine the functional relevance of spike variants emerging in immunocompromised patients, such as those identified in Case 1. Another important question is whether regular administration of REGN-COV2 or other mAb therapies may have value as primary prophylaxis against SARS-CoV-2 infection in vulnerable patient populations.

## Data Availability

Data sharing is not applicable to this article as no datasets were generated or analysed specifically for the current case report and sharing of clinical data is restricted by clinical information governance regulations.

## Abbreviations

CLL: Chronic lymphocytic leukaemia
CP: Convalescent plasma
FL: Follicular lymphoma
REGN-COV2: Casirivimab and imdevimab
vRNA: Viral RNA
XLA: X linked agammaglobulinaemia
